# Involvement of MicroRNAs in Regulation of Osteoblastic Differentiation in Mouse Induced Pluripotent Stem Cells

**DOI:** 10.1371/journal.pone.0043800

**Published:** 2012-08-24

**Authors:** Hideharu Okamoto, Yoshiaki Matsumi, Yoshiko Hoshikawa, Kazuko Takubo, Kazuo Ryoke, Goshi Shiota

**Affiliations:** 1 Division of Molecular and Genetic Medicine, Department of Genetic Medicine and Regenerative Therapeutics, Graduate School of Medicine, Tottori University, Yonago, Tottori, Japan; 2 Division of Oral and Maxillofacial Biopathological Surgery, Department of Medicine of Sensory and Motor Organs, School of Medicine, Tottori University Faculty of Medicine, Yonago, Tottori, Japan; University of Barcelona, Spain

## Abstract

**Backgoround:**

MicroRNAs (miRNAs), which regulate biological processes by annealing to the 3′-untranslated region (3′-UTR) of mRNAs to reduce protein synthesis, have been the subject of recent attention as a key regulatory factor in cell differentiation. The effects of some miRNAs during osteoblastic differentiation have been investigated in mesenchymal stem cells, however they still remains to be determined in pluripotent stem cells.

**Methodology/Principal Findings:**

Bone morphogenic proteins (BMPs) are potent activators of osteoblastic differentiation. In the present study, we profiled miRNAs during osteoblastic differentiation of mouse induced pluripotent stem (iPS) cells by BMP-4, in which expression of important osteoblastic markers such as Rux2, osterix, osteopontin, osteocalcin, PTHR1 and RANKL were significantly increased. A miRNA array analysis revealed that six miRNAs including miR-10a, miR-10b, miR-19b, miR-9-3p, miR-124a and miR-181a were significantly downregulated. Interestingly, miR-124a and miR-181a directly target the transcription factors Dlx5 and Msx2, both of which were increased by about 80-and 30-fold, respectively. In addition, transfection of miR-124a and miR-181a into mouse osteo-progenitor MC3T3-E1 cells significantly reduced expression of Dlx5, Runx2, osteocalcin and ALP, and Msx2 and osteocalcin, respectively. Finally, transfection of the anti-miRNAs of these six miRNAs, which are predicted to target Dlx5 and Msx2, into mouse iPS cells resulted in a significant increase in several osteoblastic differentiation markers such as Rux2, Msx2 and osteopontin.

**Conclusions/Significance:**

In the present study, we demonstrate that six miRNAs including miR-10a, miR-10b, miR-19b, miR-9-3p, miR-124a and miR-181a miRNAs, especially miR-124a and miR-181a, are important regulatory factors in osteoblastic differentiation of mouse iPS cells.

## Introduction

Osteoblastic differentiation is precisely regulated through activation and suppression of genes in response to physiological signals. The osteogenic bone morphogenic proteins (BMPs) are potent activators of the genes responsible for osteoblastic differentiation [1], [2]. BMPs regulate the differentiation of many cell types, including mesenchymal stem cells [3], [4]. BMP-Smad signaling, the primary pathway of osteoblastic differentiation, activates osteoblast-essential genes including the transcription factors Runx2 and osterix (OX). [5]–[7].

MicroRNAs (miRNAs) are small (22-nt) endogenous noncoding RNAs, which function at the post-transcription level by annealing to the 3′-untranslated region (3′-UTR) of target mRNAs to inhibit translation, have emerged as key regulatory factors in development, organogenesis, apoptosis, and cell proliferation and differentiation, as well as in the regulation of tumorigenesis [8], [9]. Although many miRNAs have been identified, the biological functions of relatively few have been characterized in detail. The effects of miRNAs during osteoblastic differentiation have been investigated in various cell types based on their functional regulation [10], [11], [12]. miRNA-204/211 target Runx2 production in bone marrow-derived mesenchymal stem cells (MSCs) to stimulate adipogenic differentiation and inhibit osteoblastic differentiation [13]. In mouse ST2 MSCs, miR-125b inhibited osteoblast differentiation while miR-133 and miR-135 directly targeted Runx2 and Smad5 production, inhibiting the commitment of C2C12 MSCs into bone precursor cells [14], [15]. The miRNAs miR-141 and miR-200a contribute to stimulate early osteoblastic differentiation by regulating Dlx5, their common target [16]. The miRNA miR-29b has been characterized as a positive regulatory factor because it targets inhibitors of osteoblastic differentiation, while miR-206 targets Cx43 production to inhibit osteogenesis *in vitro* and *in vivo* [17]. The role of miRNA remains to be clarified in osteoblastic differentiation in iPS cells.

**Table 1:** Sequences of primers.

Targeted gene	Sense primer(5′-3′)	Antisense primer(5′-3′)
Osteocalcin	TCTGACAAAGCCTTCATGTCC	AAATAGTGATACCGTAGATGCG
Osteopontin	TCTCCTTGCGCCACAGAATG	TCCTTAGACTCACCGCTCTT
Osterix	CCTCTGCGGGACTCAACAAC	TGCCTGGACCTGGTGAGATG
PTHR1	ACTACAGCGAGTGCCTCAAG	ACAGTCCCTCCACCAGAATC
RANKL	GGAAGCGTACCTACAGACTA	AGTACGTCGCATCTTGATCC
Runx2	CCTGAACTCTGCACCAAGTC	GAGGTGGCAGTGTCATCATC
Msx2	ATACAGGAGCCCGGCAGA	CGGTTGGTCTTGTGTTTC
Dlx5	GCCCCTACCACCAGTACG	TCACCATCCTCACCTCTG
beta-actin	GACGGCCAGGTCATCACTATTG	CCACAGGATTCCATACCCAAGA

**Table 2:** Sequneces of miRNAs for real-time RT-PCR.

	MirBase #	miRNAsequence(s)	Real-time RT-PCR primer sequence(s)
miR-124a	MIMAT0000134	UAAGGCACGCGGUGAAUGCC	TAAGGCACGCGGTGAATGC
miR-181a	MIMAT0000210	AACAUUCAACGCUGUCGGUGAGU	CATTCAACGCTGTCGGTGAGT
miR-9-3p	MIMAT0000143	AUAAAGCUAGAUAACCGAAAGU	AAAGCTAGATAACCGAAAGT
miR-10b	MIMAT0000208	UACCCUGUAGAACCGAAUUUGUG	CCCTGTAGAACCGAATTTGTGT
miR-10a	MIMAT0000648	UACCCUGUAGAUCCGAAUUUGUG	ACCCTGTAGATCCGAATTTGTG
miR-19b	MIMAT0000513	UGUGCAAAUCCAUGCAAAACUGA	GTGCAAATCCATGCAAAACTGA
U6 snRNA	NR003027.1	TGGCCCCTGCGCAAGGATG	TGGCCCCTGCGCAAGGATG

We hypothesized that miRNAs targeting the positive regulators of Wnt and BMP signaling pathways during osteoblastic differentiation are important, and we focused on those miRNAs that are downregulated during osteoblastic differentiation of mouse iPS cells. A miRNA array analysis revealed that among the miRNAs that are downregulated during osteoblastic differentiation, miR-10a, miR-10b, miR-19b, miR-9-3p, miR-124a, and miR-181a seemed most likely to target the osteogenesis-related transcription factors Dlx5 and Msx2, acting as potential inhibitors of osteogenesis by directly targeting these osteogenesis-related transcription factors. Indeed, transfection of the anti-miRNAs of these six miRNAs resulted in a significant increase in several osteoblastic differentiation markers such as Rux2, MsX2 and osteopontin, indicating the possibility that these miRNAs play an important role in the osteoblastic differentiation in mouse iPS cells.

**Figure 1:**
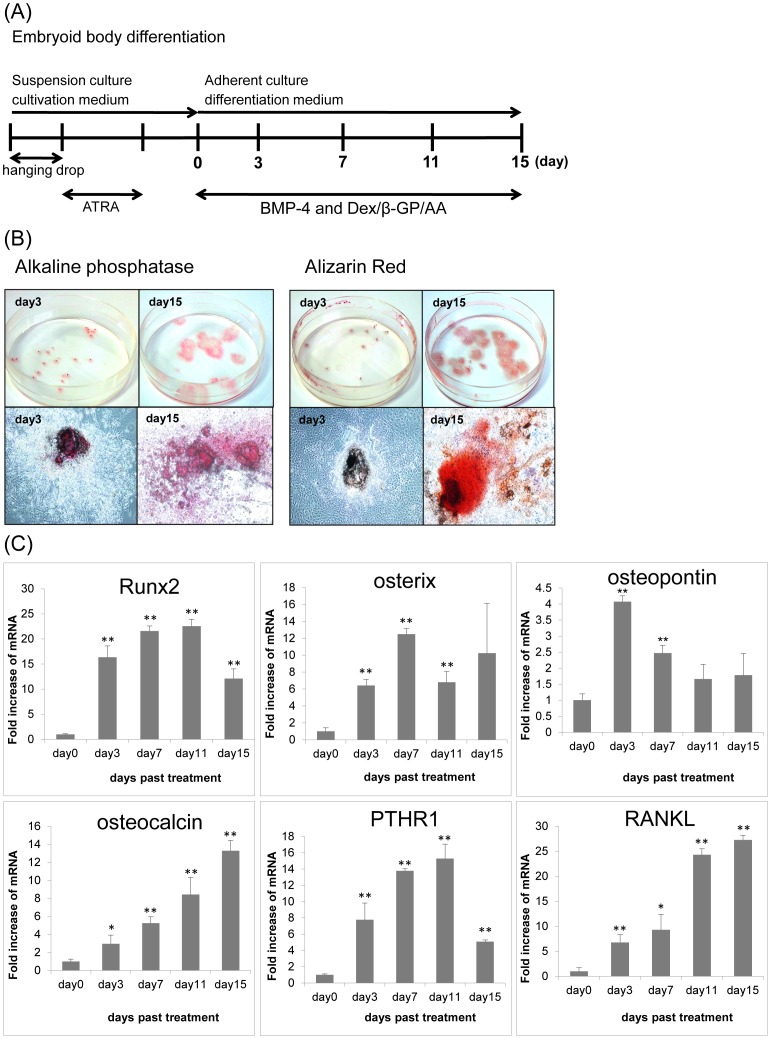
Osteoblast differentiation induced by BMP-4 in iPS cells. (A) Schematic representation of the osteoblast differentiation protocol for iPS cells by osteogenic cocktail. (B) Histochemical staining for ALP activity and with alizarin red in differentiated iPS cells on day 3 and day 15. EBs were grown for 15 days in the presence of Dex/h-GP/AA with BMP-4 and then stained (ALP and alizarin red). (C) Time course of osteoblast marker expression in osteoblastic differentiated iPS cells. Quantitative RT-PCR for osteoblast markers was carried out. Bars represent means ± SD of 3 separate wells.

## Methods

### Cell Culture

Mouse iPS cells (iPS-MEF-Ng-20D-17) [18] were maintained with feeders in Glasgow modification of Eagle medium (GMEM) supplemented with 10% fetal bovine serum (FBS), 2-mercaptoethanol, and leukemia inhibitory factor (LIF) as described elsewhere [5]. Embryoid body (EB) formation from iPS cells (iEB) and subsequent osteoblastic differentiation were carried out as reported previously [5], [19], [20]. The EBs were plated on gelatin-coated dishes for subsequent differentiation and cultured with combinations of 10 µM dexamethasone (Dex)/10 mM beta-glycerophosphate (beta-GP)/50 µg/mL ascorbic acid (AA) or 100 ng/mL BMP-4 and recombinant BMP-4 (R&D Systems, Inc., Minnesota, USA). MC3T3-E1 cells belonging to an osteo-progenitor cell line established from mouse calvaria [21], [22] were a gift from Prof. Hayashi S-I of Tottori University.

### Histochemical Staining

For histochemical detection of alkaline phosphatase (ALP), iEB outgrowths were fixed and stained using an alkaline phosphatase kit (Sigma, Missouri, USA). The iEB outgrowths were fixed with 4% paraformaldehyde in PBS and washed with distilled water (dH_2_O), and then incubated with 2% alizarin red in dH_2_O for 5 min, followed by several washes with dH_2_O.

### miRNA Microarray and Data Analysis

Five µg of total RNA extracted from differentiated iPS cells at 0, 3, 7, and 15 days were labeled using the NCode™ Rapid Labeling System (Life Technologies Corp., California, USA) and arrayed on the Multi-Species miRNA Microarray V2 (Life Technologies). Microarray procedure and data analyses were performed with the NCode miRNA Microarray (Life Technologies). Samples were labeled and hybridized in triplicate using miRNA microarray chips containing 427 mouse probes. The reported fold change represents the median fold change between the first and second tissues. Positive fold changes represented the fold increase of the second tissue over the first tissue and negative fold changes indicated fold decrease of the second tissue versus the first tissue. Fold changes will always have absolute values ≥1. Median fold changes were hierarchically clustered using Cluster and TreeView shareware programs (available from http://rana.lbl.gov/EisenSoftware.htm) for clustering analysis of normalized values and creation of a heat map of normalized values.

**Figure 2:**
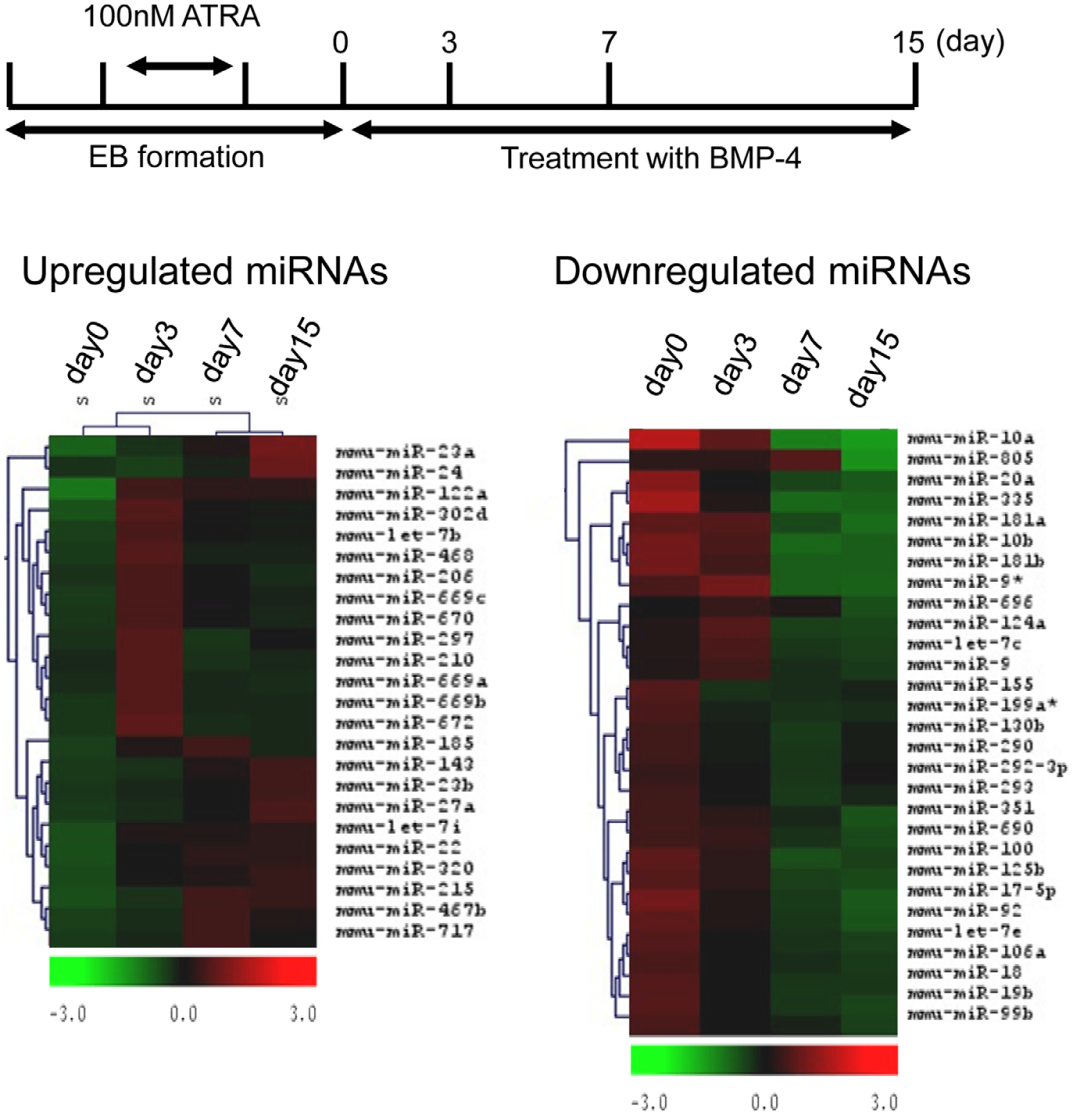
Altered expression of miRNAs during osteogenic differentiation of iPS cells. Total RNA of iPS cells during differentiation was extracted for miRNA microarray analysis at various time points (0, 3, 7, and 15 days). Relative fold changes in miRNAs were hierarchically clustered using Cluster and TreeView.

**Table 3:** Putative targets of miRNAs.

miRNA	Putative targets
miR-10a	SMAD2, DVL3, WNT8A FGF6,BMP8B, WNT6,DLX5
miR-805	FZD4, SMAD2, STAT2
miR-20a	JAK1, FZD7, COL4A4, COL4A3, MAPK14
miR-335	SAMD5, SAMD8, FZD4, MAPK6, WNT10B, DVL1, BMP7
miR-181a	TNFRSF11B(osteoprotegerin),MAPK1, BMPR2, MSX2, FOS
miR-10b	WNT8A, FGF6, BMP8B, DLX5, FGF3
miR-181b	FGF7
miR-9-3p	STAT4, ID4, DLX5, MAPK15
miR-124a	FZD4, COL4A1, BMP6, BMPR1B, MAPK14, DLX5, SP3, BMP3, DLX2
	VDR, STAT3, WNT4, STAT6
miR-351	VDR, DVL1, STAT3, MAPK14, BMPR2, MAPK12, WNT7A, DLX2, DVL3
miR-125b	MAPK14, SMAD4, BMPR2, DVL1, FGFR3, BMP3, BMP7
miR-17-5p	STAT3, MAPK4, FGF4, SMAD5, BMP2, MAPK9
miR-92	BMPR2, MAPK7
miR-19b	MAPK6, WNT9A, ID4, MSX2, PTHLH

### Quantitative RT-PCR

RNA was analyzed by quantitative RT-PCR. Briefly, TRIzol reagent (Life Technologies) was used for RNA isolation and purified total RNA was used to synthesize cDNA by reverse transcription reaction with oligo(dT) primers. Real-time RT-PCR was performed using SYBR green reagents (Roche Diagnostics GmbH, Mannheim, Germany) and mRNA levels were normalized against β-actin. The primers used for amplification are listed in Table 1. We also examined miRNA expression levels with the NCode VILO™ miRNA cDNA Synthesis Kit (Life Technologies). Briefly, cDNA was generated after reverse transcription (RT) of 500 ng total RNA, and expression levels were normalized against U6, an internal control. qRT-PCR consisted of 50 cycles (95°C for 10 s, 60°C for 40 s, and 72°C for 1 s) after an initial denaturation step (95°C for 10 min). Primers used for amplification are listed in Table 2.

**Figure 3:**
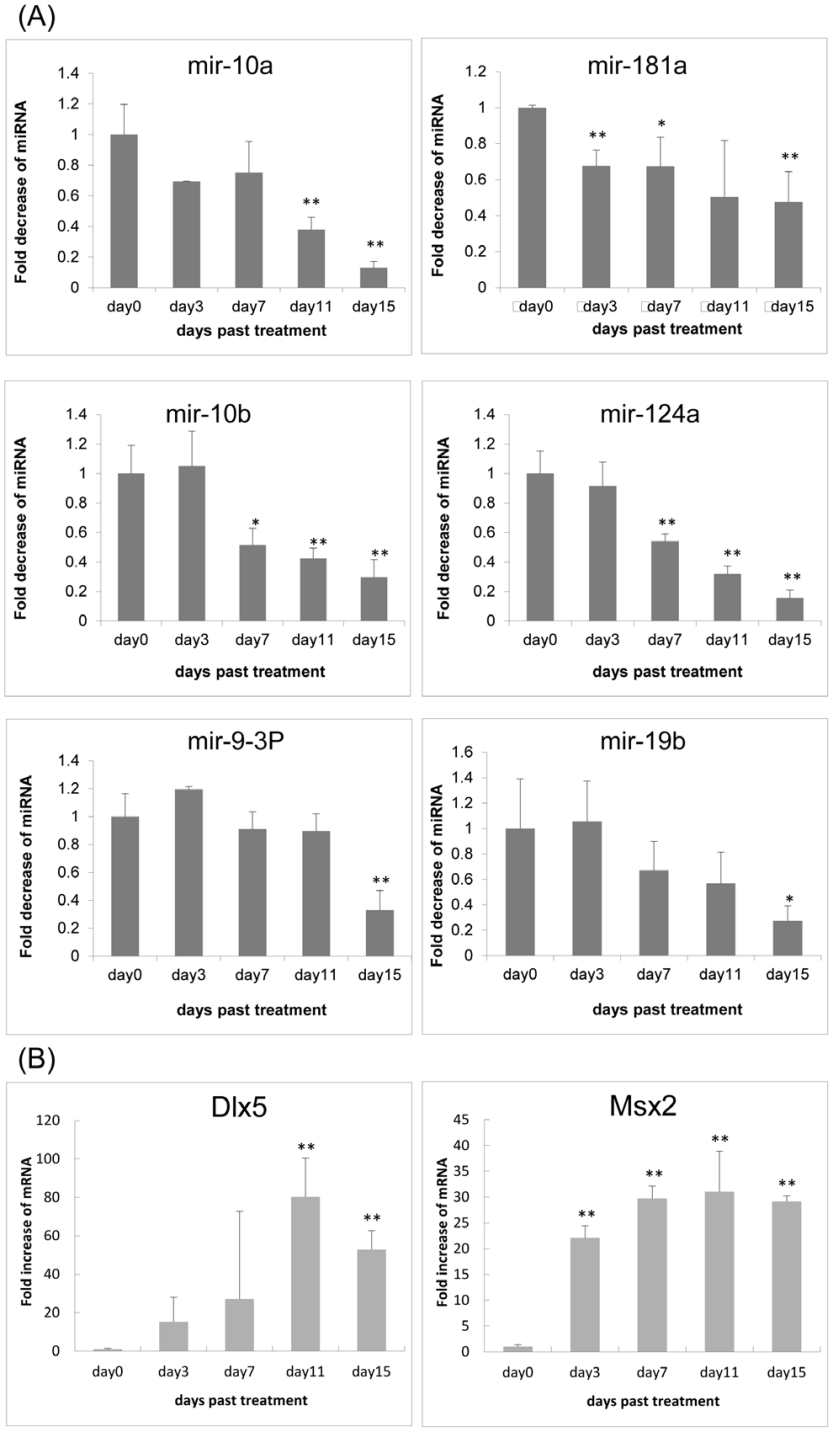
Expression of miRNAs targeting Dlx5 or Msx2 and levels of Dlx5 and Msx2 mRNA in quantitative RT-PCR. (A) Time course of miR-10a, miR-10b, miR-19b, miR-9-3p, miR-124a, and miR-181a expression in differentiated iPS cells. Quantitative RT-PCR for these miRNAs was performed. Bars represent means ± SD of 3 separate wells. (B) Time course of Dlx5 and Msx2 expression in differentiated iPS cells. Quantitative RT-PCR for osteoblast markers was performed. Values represent means ± SD of 3 separate wells.

**Figure 4:**
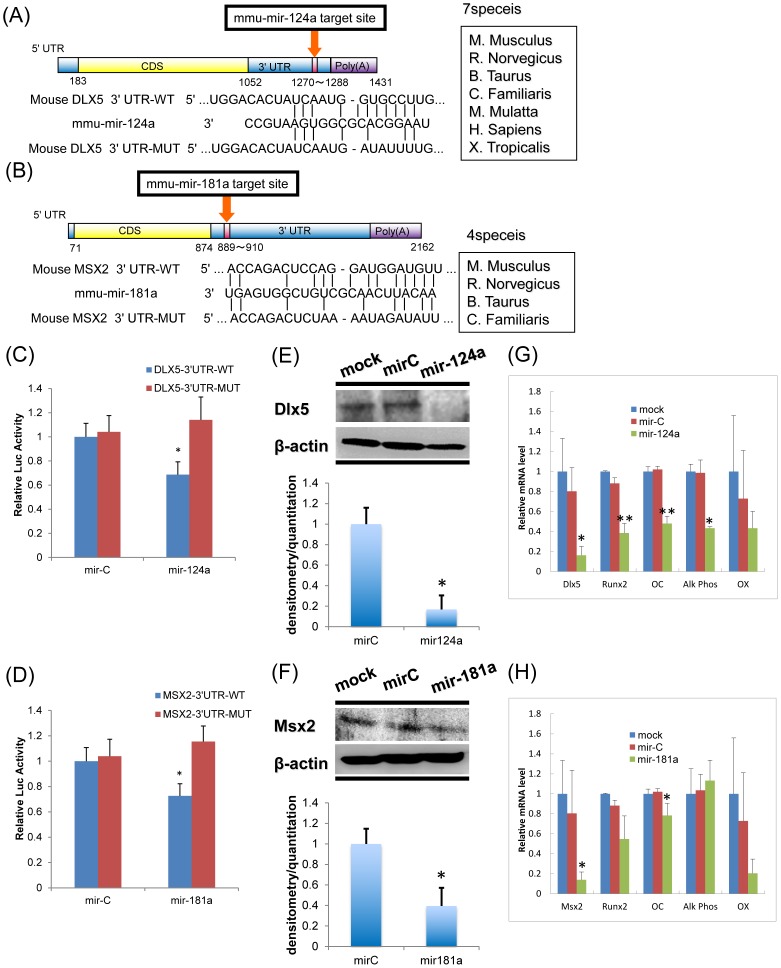
Functional activity of miR-124a and miR-181a on target genes. (A) Schematic image of miR-124a putative target site in mouse Dlx5 3′-UTR and alignment of mir-124a with wild-type (WT) and mutant (MUT) 3′-UTR regions of Dlx5 showing complementary pairing. Mutated nucleotides are underlined. These seed regions are evolutionally well-conserved among higher vertebrates. (B) Schematic image of miR-181 putative target site in the mouse Msx2 mRNA 3′-UTR and alignment of miR-181a with wild-type (WT) and mutant (MUT) 3′-UTR regions of Msx2. Complementary pairing between miR-181a and Msx2 is shown. Mutated 3′-UTR nucleotides are underlined. These seed regions are evolutionally well-conserved among higher vertebrates. (C) MC3T3 cells were cotransfected with luciferase reporters carrying wild-type Dlx5 3′-UTR or mutated Dlx5 3′-UTR, phRL-null (Renilla plasmid), and 50 nM RNA oligonucleotides of mir-Control (mir-C) or miR-124a. The effects of miR-124a and control miRNAs on reporter constructs after 36 h are shown. The ratio of reporter (Firefly) to control phRL-null plasmid (Renilla) in relative luminescence units is plotted. Values represent means ± SD of 3 separate wells. (D) Functional activity of the luciferase reporter plasmid carrying wild-type or mutated Msx2 3′-UTR was assessed as described above (Fig. 4B). Values represent means ± SD of 3 separate wells. (E) miR-124a directly targets and regulates Dlx5 and inhibits osteoblastogenesis. MC3T3 osteoblast cells were transfected with miR-124a, miRNA-Control, or transfection reagent only (Mock) at the indicated concentrations. Western blots for Dlx5 and actin (as control) were performed on total cell lysates collected at 48 h. The bands of Dlx5, Msx2 and actin were detected by ImageQuant LAS4000 (GE Healthcare UK Ltd, Little Chalfont, UK), and the intensity of bands was measured by ImageJ(http://rsb.info.nih.gov/ij/). Values represent means ± SD of 3 separate wells. *P<0.05, compared to miR-C. (F) MC3T3 cells were transfected with miR-181a and miR-Control as described in (E). Values represent means ± SD of 3 separate wells. *P<0.05, compared to miR-C. (G) Quantitative mRNA levels (normalized by β-actin) by Q-PCR for Dlx5, Runx2, ALP, OC and OX after 50 nM oligo transfection. Values represent means ± SD of 3 separate wells. * and **P<0.05 and P<0.01, compared to miR-C, respectively. (H) The mRNA levels of osteoblast marker genes after transfection of miR-Control and miR-181a were determined as described in (G). Values represent means ± SD of 3 separate wells.* and **P<0.05 and P<0.01, compared to miR-C, respectively.

### Western Blot Analysis

MC3T3-E1 osteo-progenitor cells were collected at 48 h after transfection of anti-miRNAs. Cells were lysed in lysis buffer containing protease inhibitors and 30 µg aliquots were subjected to western blot analysis using specific antibodies against Msx2 (Santa Cruz Biotechnology, California, USA), Dlx5 (Proteintech Group, Inc. Illinois, USA), and actin (Santa Cruz). Actin was used as an internal control. The bands of Dlx5, Msx2 and actin were detected by ImageQuant LAS4000 (GE Healthcare UK Ltd, Little Chalfont, UK), and the intensity of bands was measured by ImageJ(http://rsb.info.nih.gov/ij/).

**Figure 5:**
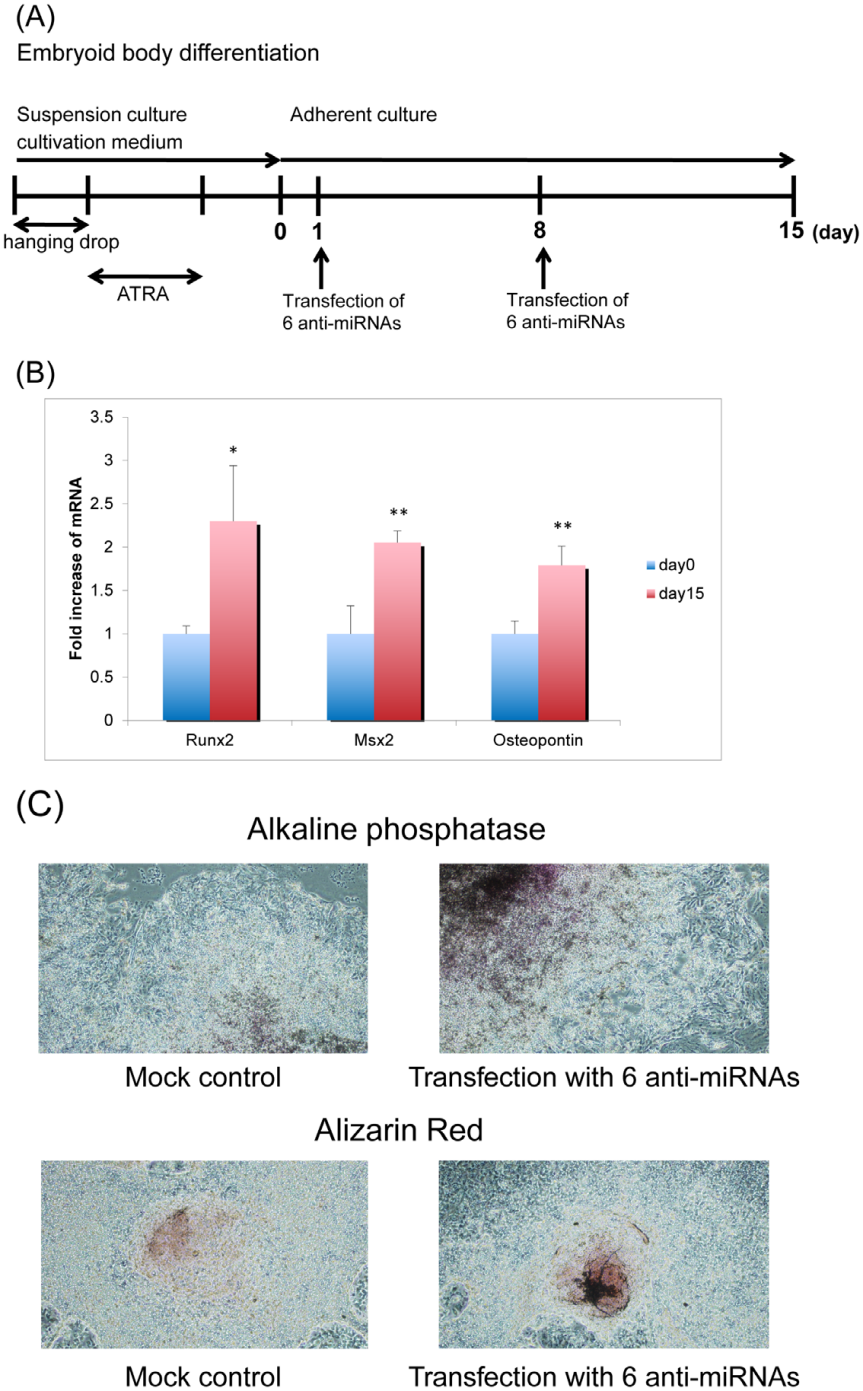
Effects of transfection with 6 anti-miRNAs on osteoblast differentiation of iPS cells. (A) Schematic representation of the osteoblast differentiation protocol for iPS cells which were transfected with 6 anti-miRNAs including anti-miR-124a, anti-miR-181a, anti-miR-10a, anti-miR-10b, anti-miR-19b, and anti-miR-9-3p. Transfection with 6 anti-miRNAs into mouse iPS cells was carried out on day 1 and day 8. Total RNA was harvested on day 15 for quantification of osteoblastic markers. (B) Expression of Runx2, Msx2 and osteopontin in mouse iPS cells on day 0 and on day 15 following transfection of 6 anti-miRNAs. The time course of the experiment follows the Fig. 5.A. Osteoblastic differentiation was examined by osteoblastic markers such as Runx2, Msx2, Dlx5, OPN, OX and OC by real-time RT-PCR. Of these markers, Runx2, Msx2 and osteopontin were significantly increased, and the other markers were not changed. Values represent means ± SD for 3 separate wells, and the data were expressed as the fold increase of mRNA on day 15 in comparison with day 0. * and **P<0.05 and P<0.01, compared to day 0, respectively. (C) Histochemical staining for ALP and alizarin red in differentiated iPS cells which were transfected with 6 anti-miRNAs. On day 15, the iPS cells which were transfected with 6 anti-miRNAs or mock control were stained (ALP and alizarin red).

### DNA Constructs

For functional analyses of miR-124 and miR-181, segments of the 3′-UTR Dlx5 and Msx2 mRNA sequences were amplified by PCR using sense and antisense primers. The products were then subcloned into the HindIII-XbaI site of a pGL3-Promoter vector (Promega Corp., Wisconsin, USA). The site mutations were confirmed by direct sequencing of PCR products.

### Reporter Assay

Double-stranded Ambion synthetic oligo-RNAs representing mature sequences that mimic the endogenous miR-124a and miR-181a miRNAs and negative control molecules were obtained from Life Technologies. Transient transfection into MC3T3-E1 cells (2.1×10^4^ cells/well) was carried out in 24-well plates with Lipofectamine 2000 (Invitrogen) in accordance with the manufacturer’s instructions. The cells were cotransfected with firefly luciferase plasmid (Promega), renilla luciferase plasmid and 50 nM microRNA, and luciferase assays were performed with a dual-luciferase reporter assay system (Promega) at 36 h after transfection in accordance with the manufacturer’s instructions. Luminescent signals were quantified with a luminometer (MiniLumat LB9506, Berthold GmbH&Co. KG, Wildbad, Germany), and each value for firefly luciferase activity was normalized by renilla luciferase activity.

### Transfection Assay

MC3T3-E1 cells at 30%–50% confluence were transfected with miR-124a, miR-181a, and the miRNA negative control, of which concentrations were 50 nM, with the oligo-fectamine product manufactured by Life Technologies in accordance with the manufacturer’s instructions. Cells were harvested at 48 h after transfection for protein and mRNA analysis. For functional studies examining the effects of the anti-miRNAs on cell differentiation, the mouse iPS cells were transfected on day 1 and day 8 after EB formation with anti-miR-124a, anti-miR-181a, anti-miR-10a, anti-miR-10b, anti-miR-19b, and anti-miR-9-3p for 72 h, followed by culture in GMEM without osteogenic factor. The effects of the anti-miRNAs on the subsequent osteoblastic differentiation were estimated by mRNA analysis and staining with ALP and alizarin red on day 15.

## Results

### Osteoblastic Differentiation of Mouse iPS Cells

Kawaguchi et al. have demonstrated that addition of BMP-4 and Dex/beta-GP/AA to EBs induced osteoblastic differentiation from embryonic stem (ES) cells [5]. We induced EBs from iPS cells, and then we induced osteoblastic differentiation with a similar protocol (Fig. 1A). The expression of osteoblast-specific markers was determined and histological staining was conducted in order to confirm osteoblastic differentiation.

The expression levels of Runx2, OX, osteopontin (OPN), osteocalcin (OC), Rankl, and Pthr1 mRNAs were determined by real-time RT-PCR. The iEBs were propagated in gelatin-coated dishes in the presence of BMP-4 and Dex/beta-GP/AA. The levels of Runx2, OX, OPN, OC, Rankl, and Pthr1 mRNA were significantly increased after 100 ng/mL BMP-4 treatment for 15 days, compared to day 0. The expression of OC, a mature osteoblast marker, increased over the differentiation process and was about 13-fold higher on day 15 than that on day 0 (Fig. 1B).

The outgrowth of iEBs was histologically stained in order to evaluate osteoblastic differentiation. The addition of BMP-4 and Dex/beta-GP/AA resulted in regions positively stained by alkaline phosphatase (ALP) and alizarin red (Fig. 1C). Alizarin red staining, which is used to detect calcium deposits, indicated the mineralization characteristics of mature osteoblasts. Real-time RT-PCR and histological staining confirmed BMP-4-induced osteoblastic differentiation.

### Downregulation of Osteogenesis-related miRNAs during BMP-4-Mediated Osteoblastic Differentiation of iPS Cells

We analyzed miRNA expression to determine which miRNAs contributed to regulation of osteoblastic differentiation. The miRNA profiling was carried out using the total RNA of the outgrowth of the iEB-induced osteoblasts from the BMP-4 treatment (Fig. 2). Total RNA was collected on days 0, 3, 7, and 15 after BMP-4 treatment. The levels of 53 miRNAs changed significantly from days 0 to 15 during the period of BMP-4-induced osteoblastic differentiation. Of these 53 miRNAs, 24 were increased and 29 were decreased. Of the 29 downregulated miRNAs, we focused on the 14 that were the most significantly (>2-fold) downregulated. Table 3 shows the putative gene targets in the microCosm and TargetScan databases of these 14 miRNAs, which seemed to play an important role in osteoblastic differentiation. These putative targets included bone regulatory proteins such as Runx2 [7], Msx2 [23], [24], Dlx3 [23], [25], Dlx5 [23], and SMAD5 [26], the Wnt/beta-catenin pathway [27], BMPs and associated receptors such as BMP-3, BMP-7 and BMPR2, JAK/STAT signaling components such as JAK1, STAT3 and STAT6, and MAPK signaling pathways such as MAPK14, and all of which have previously been implicated in promoting osteogenesis [15], [28], [29]. Thus, it appears that BMP-4 suppressed the miRNAs that target these mRNAs, helping to induce osteoblast formation. Six miRNAs including miR-10a, miR-10b, miR-19b, miR-9-3p, miR-124a, and miR-181a putatively targeted Dlx5 and Msx2 mRNA (Table 3). Thus, we followed the expression patterns of these 6 miRNAs in the iEB model of osteoblastic differentiation.

### Expression Profiles of miRNAs during Bone Phenotype Development

We focused on the 6 miRNAs, miR-10a, miR-10b, miR-19b, miR-9-3p, miR-124a, and miR-181a that were significantly downregulated during BMP-4-induced osteoblastic differentiation, and they seemed to target the transcription factors Dlx5 and Msx2 and to be associated with osteoblast differentiation (Table 3).

Real-time RT-PCR analysis showed that the expression of these miRNAs was decreased significantly over time during osteoblastic differentiation (Fig. 3A). Real-time RT-PCR analysis showed that expression of Dlx5 and Msx2 mRNA was significantly increased by about 80-and 30-fold, respectively (Fig. 3B). These findings suggest that the 6 miRNAs that putatively target the osteogenesis-related factors Dlx5 and Msx2 contributed to the regulation of differentiation of iPS cells into osteoblasts.

### mir-124a Directly Targets Dlx5, and mir-181a Directly Targets Msx2

The microCosm and TargetScan databases predicted that miR-124a and miR-181a would target Dlx5 and Msx2, respectively. The putative binding sites of miR-124a and miR-181a are the 3′-UTRs of Dlx5 and Msx2 mRNAs, respectively. These seed regions are evolutionarily well-conserved among higher vertebrates (Fig. 4A, 4B).

After determining that the putative binding region of mir-124a is located in the 3′-UTR of Dlx5 mRNA (Fig. 4A) and that that of mir-181a is located in the 3′-UTR of Msx2 mRNA (Fig. 4B), we investigated whether the miRNA binding regulates the putative targets by assessing miR-124a activity on Dlx5 and miR-181a activity on Msx2. This was performed with a reporter plasmid, into which the wild-type or mutant-type 3′-UTR binding sequences of the respective seed regions of miR-124a and miR-181a from Dlx5 and Msx2 were cloned into the 3′-UTR of a luciferase gene (Fig. 4A, 4B). Ectopic expression of miR-124a and miR-181a significantly suppressed the luciferase activity of the wild-type 3′-UTR reporter plasmids, but not that of the mutant-type 3′-UTR reporter plasmids (Fig. 4C, 4D). The functional activity of miR-124a and miR-181a was specific because the miRNA-control (miR-C) did not affect wild-type or mutant constructs. This indicated that miR-124a and miR-181a directly regulate Dlx5 and Msx2 through the 3′-UTRs of their mRNAs. The overexpression of miRNAs for the 3′-UTR wild-type and mutant-type Dlx5 and Msx2 sequences in osteoblasts confirmed that these genes are direct targets of miR-124a and miR-181a.

We introduced miR-124a and miR-181a into mouse MC3T3-E1 cells to validate the hypothesis that miR-124a and miR-181a negatively regulate osteoblastic differentiation by targeting key signal transduction factors. Transfection of miR-124a significantly downregulated endogenous Dlx5 protein expression (Fig. 4E), and it also downregulated mRNA of Dlx5, Runx2, OC and ALP, but not OX in MC3T3-E1 cells (Fig. 4G). When miR-181a was overexpressed in MC3T3-E1 cells, Msx2 protein was significantly decreased (Fig. 4F). Transfection of miR-181a also downregulated mRNA of Msx2 and OC, but not Runx2, ALP or OX in MC3T3-E1 cells (Fig. 4H). Our results show that miR-124a suppressed Dlx5 expression and miR-181a suppressed Msx2 expression, and we concluded that each miRNA significantly and negatively regulates osteoblastic differentiation.

### Promotion of Primary Osteoblast Differentiation by 6 Anti-miRNAs

Having found that miR-124a and miR-181a specifically and directly regulated and suppressed Dlx5 and Msx2, we investigated whether osteoblastic differentiation could be induced by suppression of miRNAs. The protocol shown in Fig. 5A was used to induce osteoblastic differentiation with 6 anti-miRNAs (anti-miR-124a, anti-miR-181a, anti-miR-10a, anti-miR-10b, anti-miR-9-3p, and anti-miR-19b) targeting Msx2 or Dlx5 in iPS cells. Osteoblastic differentiation was examined by osteoblastic markers such as Runx2, Msx2, Dlx5, OPN, OX and OC by real-time RT-PCR. Transfection of 6 anti-miRNAs into mouse iPS cells significantly induced expression of Runx2, Msx2 and OPN at day 15 in comparison to day 0 (Fig. 5B). However, expression of Dlx5, OX, and OC was not altered (data not shown). Osteoblastic differentiation was also evaluated with ALP and alizarin red staining. The staining of ALP or alizarin red in the iPS cells transfected with 6 anti-microRNAs was comparable to mock controls (Fig. 5C). Taken together, these findings demonstrated that these 6 anti-miRNAs plays a positive role in the primary-stage of osteoblastic differentiation from iPS cells, and may act as induction factors for osteoblastic differentiation. However, these 6 anti-miRNAs alone were not sufficient to induce bone differentiation, indicating the involvement of other factors in the regulation of osteoblastic differentiation of mouse iPS cells.

## Discussion

We used BMP-4 to selectively induce osteoblastic differentiation of iPS cells in order to characterize the regulatory mechanisms of miRNAs in osteoblastic differentiation. Previous research has shown efficient osteoblastic differentiation of ES cells with BMP-4 [5]. We hypothesized that many miRNAs that are downregulated during BMP-4-induced osteoblastic differentiation are involved in the differentiation process through inhibiting translation of numerous osteogenic mRNAs, including those that encode transcription factors, signal transduction factors, and corresponding receptors that are required for osteoblast formation. According to our findings, osteogenic programs are conducted in a tissue-specific manner, in part via many miRNAs, which are suppressed by BMP-4.

From our findings, some sets of miRNAs downregulated by BMP-4 seem to be necessary to suppress osteogenesis. In support of the notion that miRNA plays a key role in osteogenesis, recent studies have indicated that various miRNAs related to osteogenesis contribute to the differentiation of stem cells into immature osteoblasts [14]–[17]. In this study, we have demonstrated that Dlx5-or Msx2-targeted miRNAs are among those that are downregulated during BMP-4-induced osteoblastic differentiation. To our knowledge, our study is the first report to show that the annealing of miR-124a and miR-181a to Dlx5 and Msx2 mRNA reduced expression levels of these genes, inhibiting osteoblastic differentiation. Thus, the targeting of Dlx5 and Msx2 mRNA by miR-124a and miR-181a is a key mechanism for negatively regulating these factors in order to suppress osteoblastic differentiation in non-osseous cells. Dlx5 activates osteoblasts, and it is expressed in calcified regions and osteogenic surfaces, where its products regulate the expression of Runx2, OX, and OC [23], [30]–[34]. Chick and mouse osteoblast differentiation reflects elevated levels of OC expression induced by Dlx5 [23]. Msx2 promotes osteogenesis in specific cell types [23], [35], [36]. Dlx5 and Msx2 regulate the transcription activity of Runx2 [37]. These homeodomain proteins are important transcription-regulators in osteoblastic differentiation and are essential for osteogenesis because of their activation of bone-specific genes [23]. It was previously shown that miRNAs target the osteoblastic transcription factors Runx2 and Dlx5 [15], [17]. Taken together with the present results, we can infer that miRNAs regulate osteoblastic differentiation.

What are functions of these 6 miRNAs including miR-124a, miR-181a, miR-10a, miR-10b, miR-9-3p, and miR-19b in osteoblastic differentiation of mouse iPS cells? In our preliminary experiment, transfection of anti-miR-124a and anti-miR-181a did not induce osteoblastic differentiation in mouse iPS cells (data not shown), suggesting that suppression of miR-124a and miR181a, which directly target Dlx5 and Msx2, is not sufficient to induce osteoblastic differentiation of mouse iPS cells, but that suppression of at least one miRNA of miR-10a, miR-10b, miR-9-3p and miR-19b besides miR-124a and miR-181a is required for osteoblastic differentiation. Although it has been reported that a number of miRNAs, miR-204/211 [13], miR-125b [14], miR-133 and miR-135 [15], miR-141 and miR-200a [16], and miR-29b [17], were involved in osteoblastic differentiation, a few papers have been reported with regard to the functions of miR-10a, miR-10b, miR-9-3p and miR-19b. Considering the putative target genes in Table 3, miR-10a, miR-10b, miR-19b and miR-9-3p may constitute a control mechanism for Dlx5 and Msx2. In addition, miR-10a putatively targets Smad2, Wnt8A and Wnt6, and FGF6, suggesting that miR-10a reflects BMP, Wnt and FGF signals. The miRNA miR10b also may affect BMP, Wnt and FGF signals. Furthermore, miR-9-3p and miR-19b may affect JAK/STAT and MAPK pathways and MAPK and Wnt pathways, respectively. It is interesting that both miR-9-3p and miR-19b putatively target Id4, since Id4 has been reported to act as molecular switch promoting osteoblast differentiation [38]. To clarify the functions of these miRNAs, further analysis will be required.

Recent studies have shown that miRNAs contribute to cell differentiation in various tissues and cell types, including muscle, nerve, cartilage, adipose, and erythrocytes. Cardinali et al. showed that miR-221 and miR-222 were modulated during myogenesis and played a role in both the progression from myoblasts to myocytes and in the achievement of the fully differentiated phenotype [39]. Zhao et al. showed that miR-219 and miR-338 functioned in part by directly repressing negative regulators of oligodendrocyte differentiation, and that these miRNAs were important regulators of oligodendrocyte differentiation, possibly providing new targets for myelin repair [40]. Guan et al. showed that miR-365 regulated chondrocyte differentiation by directly targeting HDAC4 [41]. Karbiener et al. showed that miRNA-30c promoted human adipocyte differentiation and corepressed PAI-1 and ALK2 in hMDAS cells [42]. Wang et al. showed that following erythroid induction, miR-376a was significantly downregulated and CDK2 was released from miR-376a inhibition, thereby facilitating the escape of progenitor cells from the quiescent state into erythroid differentiation [43]. These results show the important role that miRNAs play in various differentiation mechanisms and suggest that miRNAs might coordinate larger regulatory networks.

In conclusion, our study identified osteoblast-associated miRNAs and highlighted their critical role in regulation of osteoblastic differentiation. Our data have shown that BMP-4-induced osteogenesis is associated with suppression of osteo-miRNA inhibition of common osteogenic pathways and targets that regulate osteogenesis. We found 6 miRNAs that were strongly associated and played a key role in controlling BMP-4-induced osteoblast differentiation in mouse iPS cells by suppressing the translation of their targeting genes. The nucleotide sequences of these miRNAs that were found to target Dlx5 and Msx2 could potentially be excellent candidates as osteoblastic differentiation markers for the development of drugs for treatment or prevention of disorders of osteogenesis. This study, therefore, has clinical relevance, and the creation of anti-miRNAs to induce osteoblast differentiation could spearhead the clinical application of anti-miRNA therapy.
